# More than depression: a multi-dimensional assessment of postpartum distress symptoms before and after a residential early parenting program

**DOI:** 10.1186/s12888-019-2024-8

**Published:** 2019-01-29

**Authors:** Nathan Wilson, Karen Wynter, Clare Anderson, Shanthakumar M. W. Rajaratnam, Jane Fisher, Bei Bei

**Affiliations:** 1 Faculty of Medicine, Nursing and Health Sciences, Monash Institute of Cognitive and Clinical Neurosciences, School of Psychological Sciences, Monash University, 18 Innovation Walk, Clayton Campus, Clayton, VIC 3800 Australia; 2 Global Public Health Unit, School of Public Health and Preventative Medicine, Monash University, Clayton, VIC Australia; 3 Centre for Quality and Patient Safety Research – Western Health Partnership, School of Nursing and Midwifery, Faculty of Health, Deakin University, Burwood, VIC Australia; 4 Cooperative Research Centre for Alertness, Safety and Productivity, Clayton, VIC Australia; 5 NHMRC Centre for Sleep and Circadian Neurobiology, Sydney, NSW Australia; 6 Division of Sleep and Circadian Disorders, Departments of Medicine and Neurology, Brigham and Women’s Hospital, Division of Sleep Medicine, Harvard Medical School, Boston, MA USA; 7 Masada Early Parenting Centre, Masada Private Hospital, East St Kilda, VIC Australia

**Keywords:** Postpartum, Postnatal, Depression, Anxiety, Insomnia, Impulsivity, Fatigue

## Abstract

**Introduction:**

Parents are vulnerable to psychological distress symptoms in the postpartum period. It is routine to screen for depressive symptoms, but anxiety, stress, fatigue, irritability and insomnia symptoms are less often assessed despite their prevalence. This study aimed to assess multiple dimensions of psychological distress, and their reliable change and clinically significant change among women admitted to a residential program for assistance with unsettled infant behaviors (UIB).

**Method:**

Women admitted to a five-night residential early parenting program completed self-report measures: the Depression Anxiety Stress Scale, Irritability Depression Anxiety Scale, Fatigue Severity Scale, and Insomnia Severity Index. A sub-group completed a computerized emotional Go-NoGo (EGNG) task as a measure of emotional impulsivity.

**Results:**

Seventy-eight women were recruited (*M*_*age*_ = 34.46, *SD*_*age*_ = 4.16). On admission, 48% of women reported clinically elevated depressive symptoms and 97.5% of women *not* reporting elevated depressive symptoms reported clinical elevations in at least one other form of distress. Upon discharge, all self-report distress symptoms were significantly reduced (all *p*-values <.001), but reliable and clinically significant change only occurred in a subgroup of women. There were no significant changes in indicators of impulsivity based on the EGNG.

**Conclusions:**

In addition to, and often in the absence of, depressive symptoms, women attending an early parenting program experienced a wide range of psychological distress, including fatigue, insomnia, anxiety and stress. Different forms of distress improved in different magnitudes to the treatment provided. These findings highlight the need for a multi-dimensional approach in the assessment and treatment of postpartum distress.

**Electronic supplementary material:**

The online version of this article (10.1186/s12888-019-2024-8) contains supplementary material, which is available to authorized users.

## Background

### Postpartum distress: More than depression

The postpartum period can be a time of vulnerability to distress for many women. Postpartum mental health problems are burdensome for parents, can have serious short and long-term negative impacts for infants, and cause significant costs to health care systems [1–3]. Consequently, detection and treatment of psychological distress in new motherhood is critical [4, 5]. While historically, clinical and research focus has been on maternal depression symptoms, there is growing recognition of a need to assess a broader range of psychological distress symptoms, such as anxiety, stress, fatigue, and insomnia [5–12]. These symptoms may not necessarily be identified by measures of postpartum depression [13].

While an estimated 10 to 20% of women experience postpartum depressive symptoms [14], anxiety symptoms can be equally, if not more prevalent in the post-partum period, and in many cases often co-exist with depressive symptomology [7, 10, 15, 16]. Less is known about the extent of irritability, a separate unpleasant mood state that predisposes people towards negative emotions, hostile appraisals, and the expression of negative emotions (e.g., anger) towards others [17, 18]. While irritability may exist in the context of other forms of distress such as depression, some consider it a unique construct in its own right [17, 18]. Pertinent to the postpartum periods, women may be vulnerable to irritability symptoms due to increases in sleep disturbance [11, 19, 20].

Fatigue is a common complaint in the postpartum period [21], yet to the best of our knowledge, not routinely assessed in primary or specialist healthcare settings. Fatigue is subjective feelings of overwhelming exhaustion or tiredness that reduces physical and cognitive capacities to function [22, 23], and can be caused by physical, psychological, and environmental factors [24]. Approximately 60% women report elevated fatigue in the first months after giving birth [21]. While fatigue is one of the DSM-5 diagnostic criteria for depressive disorders [25], a growing body of evidence suggests that postpartum fatigue and depression are *related but separate* constructs [9, 26–28].

Insomnia symptoms are common but have been scarcely researched in the postpartum period [8]. With 40–60% of women who have recently given birth reporting insomnia symptoms [8]. Insomnia symptoms include persistent difficulties in falling asleep, staying asleep, waking too early, or having unrefreshed sleep despite adequate sleep opportunity and conducive sleep environment, and is accompanied by impairments in daytime functioning [29]. Assessment of insomnia may assist with early detection and treatment of other forms of psychological distress such as depression, which insomnia symptoms can precede and/or perpetuate [30].

Impulsivity is another under-examined area of postpartum distress. It refers to a pattern of behavior that involves failing to inhibit rapid and unplanned responses to stimuli [31–33]. Higher impulsivity is associated with symptoms of depression, anxiety, as well as maladaptive behaviors such as suicidal behavior and substance use in adults [33, 34]. Impulsivity may be particularly relevant for women in the postpartum period as greater impulsivity has the potential to negatively impact maternal-infant relationship. For example, more impulsive parents have been found to be more critical of their children in later childhood [35]. Moreover, individuals react in a more impulsive state to negative emotional stimuli after just one night without sleep [31] which is particularly relevant to women in the postpartum period due to their increased vulnerability to sustained sleep disturbance [36–38].

### Unsettled infant behavior and vulnerability to psychological distress

Unsettled infant behavior (UIB) refers to infants’ persistent crying, resistance to settling, short sleep intervals and frequent awakenings [39]. It is reported by up to 25% of parents, has complex causes, is difficult to treat, and is distressing for parents [39, 40]. Women seeking help with UIB have reported elevated depression, anxiety, stress, fatigue and demoralization symptoms [41–43]. In Australia, there are specialist public and private residential programs that help parents manage UIB (see details in the Methods).

Past research has shown that attending residential early parenting programs that assist with the management of UIB were associated with reductions in maternal depression, anxiety, stress, fatigue and demoralization symptoms [41, 44, 45]. However, neither the extent nor change in a broad range of forms of psychological distress, including irritability, insomnia and impulsivity symptoms, has been explored in this setting.

Using both self-report measures and computerized tasks, this study aimed to characterize psychological distress symptoms in women seeking assistance with UIB, fatigue and psychological distress at a residential early parenting program, and examined reliable and clinically significant change in symptoms/scores from the start to the end of the program.

## Methods

### Participants and context

Participants were recruited from the Masada Private Hospital Early Parenting Centre (MPHEPC) in Melbourne, Australia. Participants had been referred to the unit by a medical practitioner for treatment of UIB, mild to moderate psychological distress and/or fatigue symptoms. All women admitted between June and October 2015 were invited to take part and all participants were admitted with their infants to a five-day residential program. There were no exclusion criteria. Participants in this study are a subset of a larger study conducted at this site exploring sleep, daytime functioning, and psychomotor vigilance.

Details of the intervention used at the MPHEPC are described in other publications [39, 42, 44, 46]. The intervention does not target a specific distress domain, but aims to improve maternal mental health globally. Briefly, individually tailored multidisciplinary clinical support is combined with group psychoeducational sessions in a supportive residential environment that also provides opportunities for increased maternal respite, increased sleep opportunities and supported training in infant settling skills. The intervention aims to improve recognition of infant states, infant-settling skills, foster emotional literacy in the mother-infant relationship and reduce distress by fostering a solution based focus to infant crying [42] .

Ethics approval was obtained from the Avenue Hospital Research Ethics Committee and Monash University Human Research Ethics Committee for the project and all participants provided informed consent.

### Measurements

The Depression Anxiety Stress Scale (DASS-21) is a widely used self-report measure of depression, anxiety and stress symptoms [47] previously used in postpartum settings [5, 41, 48, 49]. The stress scale of the DASS includes items such as difficulties relaxing, agitation, being over-reactive and are considered non-specific stress-related arousal [47]. Cut off scores for the depression, anxiety and stress scales are ≥5, ≥ 4 and ≥ 8 respectively. Alpha was .90 and .88 for depression, .68 and .58 for anxiety, and .78 and .74 for stress at T1 and T2 respectively.

Irritability was measured using six irritability items from the Irritability Depression Anxiety Scale (IDA-I) [50], but not the two self-harm items from the original subscale which have subsequently been shown to have poor fit [18, 51]. Respondents rate their irritability symptoms on a four-point scale, with a total irritability score ranging from 0 to 18. The IDA-I irritability subscale is a well-established measure of irritability with adequate reliability and validity [17, 18]. As there is no established cut-off for the six-item version of the scale, a conservative clinical cut-off value of IDA-I > 10, was used. Alpha for T1 was .81 and for T2 was .71.

The Fatigue Severity Scale (FSS) measures the interference of fatigue on functioning [52]. The FSS contains nine items that are averaged to range from 0 to 7, with scores ≥4 indicating clinically significant fatigue. The FSS has been used widely in non-postpartum studies and in some postpartum studies [53, 54]. Alpha for T1 and T2 were .86 and .91.

The Insomnia Severity Index (ISI) is a seven-item measure of insomnia symptom severity, satisfaction with sleep, and interference of insomnia in daily functioning [29]. Item scores are totaled, and range from 0 to 28; scores ≥15 indicate clinically significant insomnia of moderate severity [29]. Given the postpartum context, participants were asked to “not include awakenings that are directly caused by having to care for your infant or other environmental factors such as noise or light,” (i.e., sleep disruption) when rating their sleep difficulties [55]. Alpha for T1 and T2 were both .79. In addition, participants also retrospectively self-reported average total sleep time (TST) across the past week at both T1 and T2 via an item adapted from the Consensus Sleep Diary [56].

Impulsivity was measured using an objective emotional Go-NoGo task (EGNG) adapted from the longer version developed used and validated in previous studies [31, 57]. This seven-minute computer-based task involved responding as fast as possible to (“Go”) or inhibiting from responding (“NoGo”) to word stimuli. A total of 144 English words between 5 to 9 letters long were presented [31], with half being emotionally neutral (e.g. “umbrella”), and the other half emotional. The emotional words were further subdivided into positive (e.g. “happy”) and negative (e.g. “frown”) valence.

The task includes four blocks, and each block contains 72 words. The target stimuli in each block were neutral Go, neutral NoGo, emotional Go and emotional NoGo. The order of the target and non-target word stimuli presented within each block was randomized. Throughout the test, the target stimulus required of each block was displayed on the screen as a reminder. Each block lasted about 100 s, with each word presented for 300 ms and the inter-stimulus interval being 900 ms. The task was run on Windows 7 laptops with 60 Hz displays using PsychoPy software (Version 1.82.01) [58] and a keyboard was used for response input. The impulsivity indicators derived from the EGNG task are (a) hit rates (number of responses made when participants were required to not make a response) for NoGo stimuli to emotional (positive and negative) word stimuli; and (b) response times (ms) for the NoGo hits.

### Procedure

Potential participants were informed about the study prior to their admission by online advertisement on the MPHEPC website and brochures in the MPHEPC admission pack. On the day of admission all potential participants were approached by researchers and invited to participate. Participants completed paper questionnaires on the first (T1) and final (fifth; T2) day of their admission. To minimize burden, participants could choose to opt out of the objective EGNG task at T1 and T2. The EGNG task was conducted in a controlled environment when participants’ infants were asleep. Each participant first completed a practice session prior to the task at both T1 and T2. A follow up survey including the self-report measures of distress (depression, anxiety, stress, irritability, and fatigue) was posted and emailed to participants 5 weeks after discharge (T3).

### Analysis

Data preparation and initial analyses were conducted in R Version 3.3.0 [59]. For all scales, if less than 10% of items were missing, missing scale items were replaced with each participant’s mean score for other items on the scale. Paired-samples *t*-tests were conducted to compare the self-report distress symptoms between T1 and T2, with effects size measured using Cohen’s *d* (0.2 is the cut-off for a small, 0.5 a medium, and 0.8 a large effect size [60]).

Reliable Change Indices (RCI) and Clinically Significant Change indices (CSC) were also calculated [61]. The RCI is a measure of whether the magnitude of change for a participant is more than can be explained by error in measurement alone [61]. The CSC measures whether the magnitude of a change for a participant is clinically meaningful. Specifically, does the change move their score closer to the mean of a non-clinical comparison group rather than being closer to the mean of a clinical reference group [60]? The values for the CSC were compared to published clinical and non-clinical sample statistics on the DASS-21 [41, 62], IDA-I [63], ISI [55, 64], and FSS [65]. The full eight-item version of the IDA-I was used for this analysis to make comparisons with existing data. Change in the self-report data across T1, T2, and T3 was measured using robust ANOVAs, given a lack of normality [66, 67].

The EGNG results were analyzed using two-way repeated measures ANOVAs in IBM SPSS Version 24. The ANOVAs compared the main effect of TIME (T1 and T2), VALENCE (Positive and Negative) and their interactions (TIME by VALENCE) for NoGo hit rates and response times. Effect sizes were measured using partial eta squared (with 0.01 being the cut-off for a small, 0.09 a medium, and 0.25 a large effect size) [68]. We also conducted additional analyses to examine whether findings differ if relevant covariates were controlled for. Due to limited sample size, two covariates were tested: baseline depressive symptom DASS-D and maternal age. Baseline depressive symptom was chosen to represent an important part of affective state, which has been associated with impulsivity [33, 34]. Maternal age was chosen because older age has been associated with higher fatigue and depressive symptoms during the postpartum period [69–71]. Holm-Bonferroni correction was applied for all multiple comparisons in the EGNG analyses.

## Results

Eighty-five women were recruited, which represented 28% of all women admitted to the MPHEPC during the recruitment period. Of these, 78 women completed surveys at both T1 and T2, and were included in the analyses. The rate of missing data was low at < 4% for all self-report variables. Maternal and infant demographic characteristics are in Table 1. Although detailed psychiatric history was not taken, 35.9% of women recruited reported having received treatment for mental health conditions in the past. Sixty-three women agreed to participate in the EGNG tasks, with 44 completing the EGNG task at both T1 and T2. Not all participants were able to complete the task at T2 due to incompatibility of test administration with their infant’s routine. Participants who completed the EGNG were on average 2.41 years younger than those who completed self-report measures only (*p* = .01), but there were no significant differences on other demographic variables or baseline distress levels. Additionally, 31 women (40%) completed the online follow-up survey 5–12 weeks post-discharge (T3).

**Table 1:** Descriptive statistics for maternal and infant characteristics (*N* = 78)

Characteristic	*M*	*SD*	*n*	*%*
Maternal age (years)	34.46	4.16		
Infant age (months)	8.68	4.82		
Infant birth weight (kg)	3.32	0.62		
Education (≥ Tertiary level)			61	78.2
Born in Australia			53	67.9
Speak predominantly English at home			70	89.7
Living with partner (married or de-facto)			77	98.7
Previous treatment for mental health condition			28	35.9
Multiple birth (twins)			2	2.56

### Distress symptoms on admission

Results for self-report measures are in Table 2 and the percentage prevalence of symptoms above clinical cut-off scores at discharge and admission is reported in Table 3. For a correlation matrix on these measures please see Additional file 1: Table S1 in the Supplement.

**Table 2:** Descriptive statistics and change in self-report variables from T1 to T2 (*N* = 78)

	*M* (*SD*)		*t*	Cohen’s *d*	RCI			CSC
	T1 (Admission)	T2 (Discharge)			Improve	Worsen	No change	
DASS-D^a^	5.01 (4.06)	1.99 (2.07)	8.28*	0.94	38%	0%	62%	25%
DASS-A^a^	3.16 (2.63)	1.73 (1.95)	4.79*	0.54	12%	1%	87%	2%
DASS-S^a^	9.53 (3.68)	4.60 (2.22)	11.29*	1.28	49%	0%	51%	44%
IDA-I	7.18 (3.94)	2.13 (2.18)	11.76*	1.33	36%	0%	64%	35%
FSS^a^	5.17 (1.02)	4.40 (3.64)	4.84*	0.54	40%	6%	53%	29%
ISI	13.59 (5.03)	8.24 (4.43)	9.06*	1.02	45%	1%	55%	40%
TST^b^	431.9 (95.0)	453.9 (56.2)	−1.90	0.22	–	–	–	–

*Note. CSC* Clinically Significant Change, *DASS-D, DASS-A,* and *DASS-S* are Depression Anxiety Stress Scale-21 Depression, Anxiety, and Stress subscales respectively, *IDA-I* Irritability Depression Anxiety Scale – Irritability subscale, *ISI* Insomnia Severity Index, *RCI* Reliable Change Index, *TST* Total Sleep Time. RCI and CSC for IDA-I (Irritability) use the full eight item measure as per Snaith et al. 1978. ^a^(*n* = 77), ^b^(*n* = 74). *p < .001

**Table 3:** Prevalence of elevated distress symptoms on admission and discharge (*N* = 78)

Domain	Measure	Cut-off	*n* (%) Above cut-off	
			T1 (Admission)	T2 (Discharge)
Depression	DASS-D^a^	≥ 5	37 (48%)	14 (18%)
Anxiety	DASS-A^a^	≥ 4	31 (40%)	15 (19%)
Stress	DASS-S^a^	≥ 8	53 (69%)	9 (12%)
Irritability	IDA-I^b^	≥ 10	22 (28%)	0 (0%)
Fatigue	FSS^b^	≥ 4	74 (95%)	63 (82%)
Insomnia	ISI	≥ 15	37 (47%)	7 (9%)

*Note.* ^a^*n* = 77 at T1; ^b^*n* = 77 at T2; DASS-D, DASS-A, and DASS-S are Depression Anxiety Stress Scale-21 Depression, Anxiety, and Stress subscales respectively; *IDA-I* Irritability Depression Anxiety Scale – Irritability subscale, *ISI* Insomnia Severity Index

**Fig. 1:**
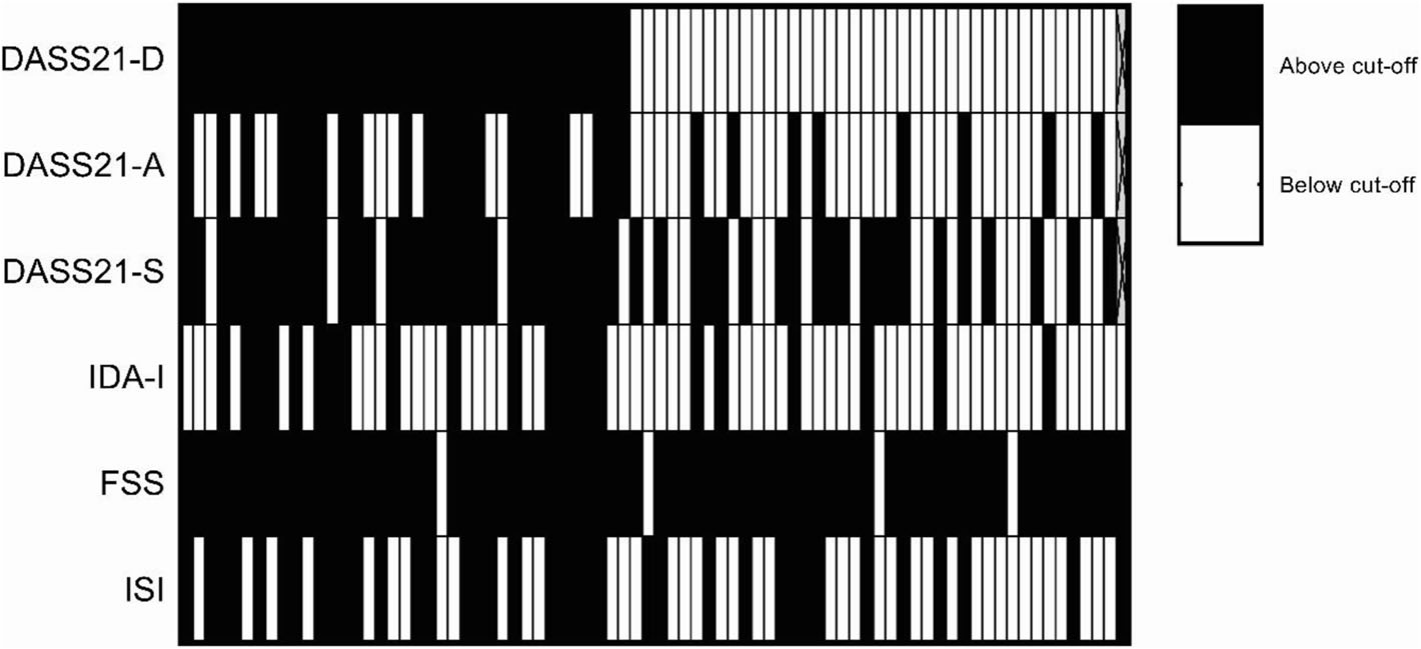
Heat map of depressive, anxiety, stress, irritability, fatigue and insomnia scores at or above clinical-cut off for women on admission with each vertical line representing one participant (*N* = 78). Heat map sorted so that women with highest DASS21-D scores are on left and lowest scores on the right. See Table 3 for cut-off values. DASS-D, DASS-A, and DASS-S are Depression Anxiety Stress Scale-21 Depression, Anxiety, and Stress subscales respectively; IDA-I = Irritability Depression Anxiety Scale – Irritability subscale; ISI = Insomnia Severity Index. Missing data was marked with crosses (*n* = 1)

On admission, fatigue was the most prevalent symptom, with nearly all (95%) women reporting fatigue symptoms above the clinical cut-off score. Stress, depressive, insomnia, anxiety and irritability symptoms above the relevant clinical cut-off scores followed fatigue in order of most to least prevalent (see Table 3). Elevation of distress scores above cut-offs across multiple domains was common, with 79% (*n* = 62) of women reporting elevation above cut-offs in two or more domains, nearly half (47%; *n* = 37) of women reporting elevation in four or more domains and 10% (*n* = 8) of women reporting elevated distress above cut-offs across all self-report symptoms. See Fig. 1 for a heat map of participants with scores above the designated clinical cut-offs for self-reported distress symptoms.

Notably, distress symptoms were common in women not reporting depressive symptoms above the clinical cut-off score. Of the 40 women not reporting elevated depressive symptoms (DASS-21D < 5), all but one participant (*n* = 39; 98%) reported symptoms above cut-off scores in at least one of the other non-depressive forms of distress assessed. With 20% (*n* = 8) of women reporting elevated anxiety symptoms, 53% (*n* = 21) elevated stress, 15% (*n* = 6) elevated irritability, 92% (*n* = 37) elevated fatigue and 33% (*n* = 13) elevated insomnia symptoms.

### Changes in psychological distress symptoms

Between admission and discharge, participants reported significant reductions in mean scores all self-reported distress related variables (see Table 2). The reductions in depression, stress, irritability and insomnia symptoms had a large effect size. The reduction in anxiety and fatigue symptoms had a medium effect size. RCI and CSC indices for changes in self-report distress symptoms between T1 and T2 are reported in Table 2.

Between T1 and T2, for depressive symptoms, 38% of women showed reliable change (improvement) above what would be expected from measurement error alone. This figure was comparable, if not higher in stress (49%), irritability (36%), fatigue (40%), and insomnia (45%). In contrast, only 12% of women experienced reliable improvements in their anxiety symptoms. Based on previously published normative data for clinical and non-clinical samples for the psychological distress variables, clinically significant change was indicated in between 25 to 44% of women. The exception again being anxiety, in which only 2% of women reported change of a magnitude that would move them from clinical to non-clinical norms (see Table 2).

The descriptive statistics for the EGNG indicators of impulsivity (hit rate and response time) for T1 and T2 are given in Table 4. There were no significant effects for TIME or VALENCE for negative or positive emotional word stimuli (all *p*-values > .05). There were also no significant interactions between TIME and VALENCE (all *p*-values > .46), with effect sizes being very small to small (range *η2 =* 0.001 to 0.021). Additional analyses were also conducted for all comparisons using depressive symptoms (DASS21-D at T1) and maternal age as covariates, with no change in the results.

**Table 4:** Emotional NoGo task hit rates and response times (*n* = 44)

Stimuli		Hit Rate		Mean Response Time (ms)	
Type	Number	T1	T2	T1	T2
Neutral	36	0.14 (0.13)	0.14 (0.16)	461 (139)	447 (124)
Emotional	36	0.26 (0.17)	0.22 (0.17)	462 (126)	479 (135)
Positive	18	0.26 (0.21)	0.20 (0.17)	445 (110)	494 (163)
Negative	18	0.26 (0.19)	0.25 (0.21)	479 (138)	467 (107)
TOTAL	72	0.20 (0.16)	0.18 (0.17)	462 (135)	467 (131)

*Note.* Means (standard deviations) are presented. T1 = admission; T2 = discharge; Hit rate: incorrect false positive responses to presented stimuli with 1.00 equal to 100% incorrect responses

### Follow-up

The post-discharge follow-up scores for self-reported depression, anxiety, stress, irritability and fatigue were compared across T1, T2 and T3 for participants that completed measures for all time points (*n* = 31) using robust ANOVAs (all *p* <. 05). Post-hoc comparisons between T2 and T3 demonstrated that there were no significant differences between depression (*psihat* = .12, *p* = .74), anxiety (*psihat* = .50, *p* = .16), stress (*psihat* = −.17, *p* = .78), irritability (*psihat* = −.50, *p* = .40) and fatigue (*pshihat* = 0.31, *p* = .21) scores.

## Discussion

Using a multi-dimensional approach, this study examined the nature and change in a range of psychological distress symptoms among women admitted to a residential early parenting program. These women experienced high a level of morbidity for psychological distress symptoms. Most women (79%) reported clinical elevation in two or more domains of distress. At discharge, all self-reported distress symptoms reduced significantly, but reduced to different extents. Reliable change or clinically significant change occurred in only a subset of women. There was no evidence of elevated impulsivity or change in emotional impulsivity from admission to discharge based on the computerized EGNG task. For participants who completed follow-up survey, the reductions in distress were sustained in the weeks after discharge.

### Postpartum distress has many dimensions

Consistent with previous studies in similar postpartum populations, women in this study not only reported elevated depressive symptoms, but also anxiety and stress symptoms [41, 46]. Adding to the current literature, we found that although nearly half of the sample reported low depressive symptoms, almost all (97.5%) of these women reported elevation in another form of psychological distress, most commonly fatigue, followed by stress. This highlights that postpartum distress is consisted of a constellation of different distress symptoms, and that depression alone does not capture its multi-dimensional nature.

It is also worth noting that on admission, stress symptoms were higher compared to depressive or anxiety symptoms, which is also consistent with previous studies in similar populations [41, 43] and in the general adult community [62]. This suggests that non-specific stress-related arousal as measured by the DASS may be a prominent part of the emotional experiences for women attending this program at the MPHEPC. Identification of stress is important, as it may co-exist with postpartum fatigue symptoms (stress and fatigue correlated at *r* = 0.67 at baseline in this study). Elevated irritability is also noted, with a third of women also reporting high levels of irritability (IDA ≥ 10). There is scant normative data for irritability in the postpartum period, but it was noted that 40 % of women endorsed an item related to getting angry with themselves and 73% reported that they lost their temper and shouted at others.

Fatigue was a near universal experience in the studied sample, and likely a significant contributing factor to women seeking admission at this residential early parenting program. In our sample 95% of women reported fatigue levels above the clinical cut-off established among chronic illness populations on the FSS. Average levels of fatigue were also higher in this study than those reported in postpartum community samples that have used the FSS [53, 72].

The extent of insomnia symptoms was also high, with nearly half of the participants reporting clinically significant insomnia symptoms. This prevalence is consistent with the only study of insomnia rates among women in the community during the postpartum period [8]. In this study, on admission insomnia symptoms strongly correlated with stress (*r* = 0.72), suggesting that non-specific arousal symptoms are increasing with greater sleep difficulties and concerns related to the interference of sleep disturbance. Alternatively, increased stress-related arousal may be making it more difficult for some women to get to sleep. These elevated insomnia symptoms most commonly co-occurred with fatigue symptoms followed by stress symptoms (see Fig. 1).

For the EGNG task at T1 and T2 mean hit rates were higher and more variable (i.e., greater *SD*) and mean response times were slower and more variable than those reported by young males in fully rested control (e.g. uninterrupted sleep) and in sleep deprivation conditions (Anderson & Platten, 2011). These differences in hit rates and response times may be due to a combination of different types of sleep disturbance experienced (sleep deprivation in past laboratory studies vs. sleep fragmentation in postpartum women), gender differences in response to sleep disturbance, differences in the computer platforms used to run the task and differences in the task duration [73–77]; although longer task durations are more likely to worsen performance, including more variability [73].

### Change in distress symptoms

The finding that attending a residential early parenting program was associated with significant reductions in maternal fatigue, depressive, anxiety and stress symptoms is consistent with findings from past studies of residential early parenting programs [41, 44, 45, 78] as well as community based infant settling/sleep intervention [79]. Extending the current literature, this study demonstrated that attendance at an early parenting program is associated with reliable and clinically significant reductions in fatigue, insomnia and irritability symptoms among a sizable proportion of the women who attended. It is worth noting that although the residential program did not target depression or anxiety specifically, effect sizes in pre-post change in this study are comparable to those observed in interventions that are tailored to these conditions [80]. This suggests that in the current population, an intervention that uses a non-specific approach to addresses postpartum distress and unsettled infant behaviours may lead to meaningful improvements in specific domains.

The effect sizes for reduction in stress, irritability and insomnia symptoms were larger than those observed for depression, and substantially larger than those for the anxiety and fatigue symptoms. Overall, this highlights that different forms of psychological distress responded in different magnitude to the intervention provided at the MPHEPC. Non-specific stress arousal, irritability, and insomnia symptoms may be particularly responsive to the intervention provided.

Furthermore, the presence of a medium effect size in reduction in fatigue symptoms and reliable change in fatigue symptoms for 40% of participants is higher than those reported in previous intervention studies that target specifically postpartum fatigue, where minimal reductions of fatigue were reported [53, 81]. As experiences of stress, fatigue, irritability and insomnia may all be sensitive to sleep disturbance [20, 77, 82, 83], it is possible that the respite opportunities and increased maternal sleep opportunities provided at the MPHEPC, may underlie these improvements.

It is also noted that only a minority of participants experienced either reliable or clinically significant change in all distress symptoms at discharge, even though there were significant mean reductions in most symptoms. The results at discharge also highlight the persistent nature of fatigue, as despite the significant reductions in mean fatigue levels, 82% of participants still reported residual fatigue above the cut-off for the FSS. As women participating in the program had infants that were on average over 8 months old, it is possible that they have been experiencing persistent sleep disturbance, fatigue and other distress symptoms for many months. The short five-day residential program may not be sufficient to achieve clinically significant change in a majority of women given the duration of these difficulties.

### Limitations and strengths

This study recruited from an ongoing clinical service and did not involve a control intervention. Whilst findings are clinically informative, it is not possible to attribute the changes seen in distress symptoms directly to the residential program or its specific components. We also did not assess infant behavior and their potential contributions to maternal distress symptoms. Also, the recruitment rate was modest, but distress or demographic characteristics were comparable to a larger study conducted at the same site with a higher recruitment rate [41]. The poor retention rate for the follow up data (T3) and variability in timing of the completion of the follow up survey allows us to make only preliminary conclusions about how benefits were maintained in the longer-term.

Women who completed the EGNG (mean age 33.4) were on average 2.4 years younger than those who completed questionnaire only (mean age 35.8). It is not clear whether this difference would have meaningful impact on a task such as the EGNG in fully developed adult women, and findings on EGNG may not generalize to other age groups.

A further limitation was the low internal consistency of DASS-21 anxiety subscale, which is under the threshold of 0.7 for being considered acceptable for both time points [84]. Responses to this scale appeared to have a floor effect. Although past postpartum samples have reported adequate internal consistency for this scale [5, 48], poor reliability and substantial floor effects similar to those in this study were noted by other studies in community adult and chronic pain populations [85, 86]. Consequently, results related to DASS anxiety in this study need to be interpreted with caution.

There is limited research on impulsivity, and it is not clear how findings based on EGNG may be comparable or interpreted in the same manner as questionnaire-based measures of impulsivity. Finally, time-of-day related differences in administration of the EGNG task between admission and discharge may have also influenced the results due to associated variations in sleepiness [87]. However, the sensitivity of the EGNG task to time-of-day effects has not been tested, and the average difference in administration times was minimal: mean time of computerized testing was 55 min (*SD* = 141) earlier at T2 (*p* < .05) compared to T1 (15:42 PM vs. 14:47 PM).

Despite these limitations, this is, to the best of our knowledge, the first study that assessed a wide range of distress symptoms that includes insomnia in a sample of women seeking clinical support in the postpartum period, and documented the changes in symptoms after attending a program that helps manage UIB. Methodological strengths included the use of both self-report and computerized neurobehavioral measures, assessing important domains that are not well examined in the existing literature (e.g., irritability, insomnia, fatigue), and using reliable and clinically significant indicators of change to facilitate interpretation.

## Conclusions and implications

It is routine practice in many postpartum clinical settings and in research studies to assess maternal depressive symptoms [6, 88]. However, the focus on postpartum depression may mean that other types of psychological distress are potentially overlooked [7]. Our study found that in addition to depressive symptoms, women experienced elevated stress, fatigue, irritability and insomnia symptoms, similar to past studies in similar settings that reported elevations of non-depressive symptoms [43, 46]. Notably, a sizable proportion of women who did not report elevated depressive symptoms, in fact, reported other psychological distress symptoms that may require clinical attention. Routine screening for postpartum depression symptoms is not likely to identify the breadth of the fatigue, stress, irritability and insomnia symptoms experienced by these women. Given the prevalence of UIB [42], it is likely that many other women in the community are experiencing similar symptoms that are under-recognized.

Our findings echo the rising recognition of the need to assess a broader range of postpartum psychological distress symptoms in primary and specialist care settings alongside postpartum depression [5, 7, 9, 11]. Currently, the relative lack of awareness of other types of distress such as fatigue, insomnia and non-specific stress among health-care practitioners may be a barrier to more comprehensive assessment [4]. Furthermore, although promising scales are being developed and validated for postpartum fatigue and anxiety [89–92], the current lack of reliable and valid measures for different types of postpartum distress symptoms is a barrier to routine assessment in a broader range of healthcare settings in the community. Validation of a brief measure covering several domains may be helpful, such as the Self-Rated Level 1 Cross-Cutting Symptom Measure being trialed with DSM-5 [25].

A multi-dimensional assessment of postpartum distress is important as it could lead to timely, and better tailored treatment of maternal distress. Women in the postpartum period with elevated fatigue, stress, and insomnia could potentially benefit from existing evidence-based interventions such as stress reduction, fatigue management, and cognitive behavior therapy for insomnia [53, 81, 93, 94]. As in our study, various forms of distress did not respond equally to the same treatment, assessing and targeting specific residual symptoms may enhance outcomes.

Finally, results in this study showed that attending a multi-disciplinary residential early parenting program has the potential to reduce a wide range of postpartum distress symptoms. Future studies, especially those that include careful measurements of infant and partner [95] related factors, are needed to better understand mechanisms associated with these changes and how they could be adopted more broadly.

## Additional file

Additional file 1: **Table S1.** Pearson Correlations for Self-Report Variables at T1 and T2 (*N* = 78). (DOC 47 kb)

## Abbreviations

CSC: Clinically Significant Change
DASS-21: Depression Anxiety Stress Scale (21)
EGNG: Emotional Go-NoGo Task
FSS: Fatigue Severity Scale
IDA-I: Irritability Depression Anxiety Scale – Irritability Subscale
ISI: Insomnia Severity Index
RCI: Reliable Change Index
RT: Response Time
T1: Time 1 (Admission)
T2: Time 2 (Discharge)
T3: Time 3 (Follow up)
TST: Total Sleep Time
UIB: Unsettled Infant Behaviour

## References

[1] Field T (2010). Postpartum depression effects on early interactions, parenting, and safety practices: a review. Infant Behav Dev.

[2] Matijasevich A, Murray J, Cooper PJ, Anselmi L, Barros AJD, Barros FC, Santos IS (2015). Trajectories of maternal depression and offspring psychopathology at 6 years: 2004 Pelotas cohort study. J Affect Disord.

[3] Petrou S, Cooper P, Murray L, Davidson LL (2002). Economic costs of post-natal depression in a high-risk British cohort. Br J Psychiatry.

[4] Hayes BA (2010). From 'postnatal depression' to 'perinatal anxiety and depression': key points of the National Perinatal Depression Plan for nurses and midwives in Australian primary health care settings. Contemp Nurse.

[5] Miller RL, Pallant JF, Negri LM (2006). Anxiety and stress in the postpartum: is there more to postnatal distress than depression?. BMC Psychiatry.

[6] Milgrom J, Gemmill AW (2014). Screening for perinatal depression. Best Pract Res Clin Obstet Gynaecol.

[7] Wynter K, Rowe H, Fisher JRW (2013). Common mental disorders in women and men in the first six months after the birth of their first infant: a community study in Victoria, Australia. J Affect Disord.

[8] Sivertsen B, Hysing M, Dørheim SK, Eberhard-Gran M (2015). Trajectories of maternal sleep problems before and after childbirth: a longitudinal population-based study. BMC Pregnancy Childbirth.

[9] Giallo R, Gartland D, Woolhouse H, Brown S (2015). Differentiating maternal fatigue and depressive symptoms at six months and four years post partum: considerations for assessment, diagnosis and intervention. Midwifery.

[10] Fairbrother N, Young AH, Janssen P, Antony MM, Tucker E (2015). Depression and anxiety during the perinatal period. BMC Psychiatry.

[11] Williamson JA, O'Hara MW, Stuart S, Hart KJ, Watson D (2015). Assessment of postpartum depressive symptoms: the importance of somatic symptoms and irritability. Assessment.

[12] Wynter K, Wilson N, Bei B, Thean P, Fisher J. Psychological and sleep-related functioning among women with unsettled infants in Victoria, Australia: A cross-sectional study. J Reprod Infant Psychol. 2018. 10.1080/02646838.2018.1556787. 10.1080/02646838.2018.1556787 30569753

[13] Rowe HJ, Fisher JRW, Loh WM (2008). The Edinburgh postnatal depression scale detects but does not distinguish anxiety disorders from depression in mothers of infants. Arch Womens Ment Health.

[14] Putnam KT, Wilcox M, Robertson-Blackmore E, Sharkey K, Bergink V, Munk-Olsen T, Deligiannidis KM, Payne J, Altemus M, Newport J (2017). Clinical phenotypes of perinatal depression and time of symptom onset: analysis of data from an international consortium. Lancet Psychiatry.

[15] Falah-Hassani K, Shiri R, Dennis C-L (2016). Prevalence and risk factors for comorbid postpartum depressive symptomatology and anxiety. J Affect Disord.

[16] Reck C, Struben K, Backenstrass M, Stefenelli U, Reinig K, Fuchs T, Sohn C, Mundt C (2008). Prevalence, onset and comorbidity of postpartum anxiety and depressive disorders. Acta Psychiatr Scand.

[17] Craig KJ, Hietanen H, Markova IS, Berrios GE (2008). The irritability questionnaire: a new scale for the measurement of irritability. Psychiatry Res.

[18] Snaith RP, Taylor CM (1985). Irritability: definition, assessment and associated factors. Br J Psychiatry.

[19] Bowen A, Bowen R, Balbuena L, Muhajarine N (2012). Are pregnant and postpartum women moodier? Understanding perinatal mood instability. J Obstet Gynaecol Can.

[20] Kamphuis J, Meerlo P, Koolhaas JM, Lancel M (2012). Poor sleep as a potential causal factor in aggression and violence. Sleep Med.

[21] McGovern P, Dowd B, Gjerdingen D, Dagher R, Ukestad L, McCaffrey D, Lundberg U (2007). Mothers' health and work-related factors at 11 weeks postpartum. Ann Fam Med.

[22] Aaronson LS, Teel CS, Cassmeyer V, Neuberger GB, Pallikkathayil L, Pierce J, Press AN, Williams PD, Wingate A (1999). Defining and measuring fatigue. Image J Nurs Sch.

[23] Shen J, Barbera J, Shapiro CM (2006). Distinguishing sleepiness and fatigue: focus on definition and measurement. Sleep Med Rev.

[24] Milligan RA, Lenz ER, Parks PL, Pugh LC, Kitzman H (1996). Postpartum fatigue: clarifying a concept. Sch Inq Nurs Pract.

[25] American Psychiatric Association: Diagnostic and Statistical Manual of Mental Disorders (5th ed.). Washington, DC: American Psychiatric Pub; 2013.

[26] Giallo R, Wade C, Cooklin A, Rose N (2011). Assessment of maternal fatigue and depression in the postpartum period: support for two separate constructs. J Reprod Infant Psychol.

[27] Wilson N, Wynter K, Fisher J, Bei B (2018). Related but different: distinguishing postpartum depression and fatigue among women seeking help for unsettled infant behaviours. BMC Psychiatry.

[28] Wilson N, Lee JJ, Bei B (2019). Postpartum fatigue and depression: a systematic review and meta-analysis. J Affect Disord.

[29] Bastien CH, Vallières A, Morin CM (2001). Validation of the insomnia severity index as an outcome measure for insomnia research. Sleep Med.

[30] Cheuk Yan S, Wong WS (2011). The effect of optimism on depression: the mediating and moderating role of insomnia. J Health Psychol.

[31] Anderson C, Platten CR (2011). Sleep deprivation lowers inhibition and enhances impulsivity to negative stimuli. Behav Brain Res.

[32] Horn NR, Dolan M, Elliott R, Deakin JFW, Woodruff PWR (2003). Response inhibition and impulsivity: an fMRI study. Neuropsychologia.

[33] Morean ME, DeMartini KS, Leeman RF, Pearlson GD, Anticevic A, Krishnan-Sarin S, Krystal JH, O'Malley SS (2014). Psychometrically improved, abbreviated versions of three classic measures of impulsivity and self-control. Psychol Assess.

[34] Tomko RL, Lane SP, Pronove LM, Treloar HR, Brown WC, Solhan MB, Wood PK, Trull TJ (2015). Undifferentiated negative affect and impulsivity in borderline personality and depressive disorders: a momentary perspective. J Abnorm Psychol.

[35] Griggs MS, Mikami AY (2011). Parental attention-deficit/hyperactivity disorder predicts child and parent outcomes of parental friendship coaching treatment. J Am Acad Child Adolesc Psychiatry.

[36] Bei B, Coo S, Trinder J (2015). Sleep and mood during pregnancy and the postpartum period. Sleep Med Clin.

[37] Insana SP, Williams KB, Montgomery-Downs HE (2013). Sleep disturbance and neurobehavioral performance among postpartum women. Sleep.

[38] Wilson N, Wynter K, Anderson K, Rajaratnam SMW, Fisher J, Bei B. Postpartum fatigue, daytime sleepiness, and psychomotor vigilance are modifiable through a brief residential early parenting program. Sleep Med (In Press). 10.1016/j.sleep.2019.01.012 31158786

[39] Fisher JRW, Rowe H, Hiscock H, Jordan B, Bayer J, Colahan A, Amery V. Understanding and responding to unsettled infant behaviour. Australian Research Alliance for Children and Youth. 2011;1–60. https://trove.nla.gov.au/work/151577056?q&versionId=165233214.

[40] Douglas PS, Hill PS, Brodribb W (2011). The unsettled baby: how complexity science helps. Arch Dis Child.

[41] Bobevski I, Rowe H, Clarke DM, McKenzie DP, Fisher JRW (2015). Postnatal demoralisation among women admitted to a hospital mother-baby unit: validation of a psychometric measure. Arch Womens Ment Health.

[42] Fisher JRW, Feekery C, Rowe H. Psycho-educational Early Parenting Interventions to Promote Infant Mental Health. In: Fitzgerald HE, Puura K, Tomlinson M, Paul C, editors. International Perspectives on Children and Mental Health. Santa Barbara: ABC-CLIO; 2011. p. 205–36.

[43] Giallo R, Rose N, Vittorino R (2011). Fatigue, wellbeing and parenting in mothers of infants and toddlers with sleep problems. J Reprod Infant Psychol.

[44] Fisher JRW, Feekery C, Rowe H (2004). Treatment of maternal mood disorder and infant behaviour disturbance in an Australian private mothercraft unit: a follow-up study. Arch Womens Ment Health.

[45] Matthey S, Speyer J (2008). Changes in unsettled infant sleep and maternal mood following admission to a parentcraft residential unit. Early Hum Dev.

[46] Fisher JR, Feekery CJ, Rowe-Murray HJ (2002). Nature, severity and correlates of psychological distress in women admitted to a private mother-baby unit. J Paediatr Child Health.

[47] Lovibond PF, Lovibond SH (1995). Manual for the depression, anxiety and stress scales (DASS).

[48] Bei B, Milgrom J, Ericksen J, Trinder J (2010). Subjective perception of sleep, but not its objective quality, is associated with immediate postpartum mood disturbances in healthy women. Sleep.

[49] Cunningham NK, Brown PM, Brooks J, Page AC (2013). The structure of emotional symptoms in the postpartum period: is it unique?. J Affect Disord.

[50] Snaith RP, Constantopoulos AA, Jardine MY, McGuffin P (1978). A clinical scale for the self-assessment of irritability. Br J Psychiatry.

[51] Maltby J, Dale M, Underwood M, Simpson J, the RiotEHsDN. Irritability in Huntington's disease: factor analysis of Snaith's irritability scale. Mov Disord Clin Pract. 2017;4(3):342–48. 10.1002/mdc3.12424 PMC6174383 30363422

[52] Krupp LB, LaRocca NG, Muir-Nash J, Steinberg AD (1989). The fatigue severity scale. Application to patients with multiple sclerosis and systemic lupus erythematosus. Arch Neurol.

[53] Giallo R, Cooklin A, Dunning M, Seymour M: The Efficacy of an Intervention for the Management of Postpartum Fatigue. In*.*; 2014. 10.1111/1552-6909.12489 25139257

[54] Shahid A, Shen J, Shapiro CM (2010). Measurements of sleepiness and fatigue. J Psychosom Res.

[55] Swanson LM, Pickett SM, Flynn H, Armitage R (2011). Relationships among depression, anxiety, and insomnia symptoms in perinatal women seeking mental health treatment. J Women's Health.

[56] Carney CE, Buysse DJ, Ancoli-Israel S, Edinger JD, Krystal AD, Lichstein KL, Morin CM (2012). The consensus sleep diary: standardizing prospective sleep self-monitoring. Sleep.

[57] Murphy FC, Smith KA, Cowen PJ, Robbins TW, Sahakian BJ (2002). The effects of tryptophan depletion on cognitive and affective processing in healthy volunteers. Psychopharmacology.

[58] Peirce JW (2007). PsychoPy--psychophysics software in python. J Neurosci Methods.

[59] Team RC (2017). R: a language and environment for statistical computing.

[60] Cohen J (1988). Statistical power analysis for the behavioral sciences.

[61] Morley S, Dowzer CN (2014). Manual for the Leeds reliable change Indicator: simple excel(tm) applications for the analysis of individual patient and group data.

[62] Crawford J, Cayley C, Lovibond PF, Wilson PH, Hartley C (2011). Percentile norms and accompanying interval estimates from an Australian general adult population sample for self-report mood scales (BAI, BDI, CRSD, CES-D, DASS, DASS-21, STAI-X, STAI-Y, SRDS, and SRAS). Aust Psychol.

[63] Condon JT, Boyce P, Corkindale CJ (2004). The first-time fathers study: a prospective study of the mental health and wellbeing of men during the transition to parenthood. Aust N Z J Psychiatry.

[64] Swanson LM, Flynn H, Adams-Mundy JD, Armitage R, Arnedt JT (2013). An open pilot of cognitive-behavioral therapy for insomnia in women with postpartum depression. Behav Sleep Med.

[65] Valko PO, Bassetti CL, Bloch KE, Held U, Baumann CR (2008). Validation of the fatigue severity scale in a Swiss cohort. Sleep.

[66] Wilcox RR. Introduction to robust estimation and hypothesis testing. San Diego: Academic Press; 2012.

[67] Field A, Miles J, Field Z. Discovering statistics using R. London: Sage publications; 2012.

[68] Pallant J (2013). SPSS survival manual: a step by step guide to data analysis using IBM SPSS (5. Uppl.).

[69] Troy NW, Dalgas-Pelish P (1997). The natural evolution of postpartum fatigue among a group of primiparous women. Clin Nurs Res.

[70] Wambach KA (1998). Maternal fatigue in breastfeeding primiparae during the first nine weeks postpartum. J Hum Lact.

[71] Upadhyay RP, Chowdhury R, Aslyeh S, Sarkar K, Singh SK, Sinha B, Pawar A, Rajalakshmi AK, Kumar A (2017). Postpartum depression in India: a systematic review and meta-analysis. Bull World Health Organ.

[72] Giallo R, Seymour M, Dunning M, Cooklin A, Loutzenhiser L, McAuslan P (2015). Factors associated with the course of maternal fatigue across the early postpartum period. J Reprod Infant Psychol.

[73] Basner M, Mollicone D, Dinges DF (2011). Validity and sensitivity of a brief psychomotor vigilance test (PVT-B) to Total and partial sleep deprivation. Acta Astronaut.

[74] Fatima Y, Doi SAR, Najman JM, Mamun AA (2016). Exploring gender difference in sleep quality of Young adults: findings from a large population study. Clin Med Res.

[75] Khitrov MY, Laxminarayan S, Thorsley D, Ramakrishnan S, Rajaraman S, Wesensten NJ, Reifman J (2014). PC-PVT: a platform for psychomotor vigilance task testing, analysis, and prediction. Behav Res Methods.

[76] Van Dongen HPA, Maislin G, Mullington JM, Dinges DF (2003). The cumulative cost of additional wakefulness: dose-response effects on neurobehavioral functions and sleep physiology from chronic sleep restriction and total sleep deprivation. Sleep.

[77] Montgomery-Downs HE, Stremler R, Insan SP (2013). Postpartum sleep in new mothers and fathers. Open Sleep J.

[78] Fisher JRW, Rowe H, Feekery C (2004). Temperament and behaviour of infants aged 4–12 months on admission to a private mother-baby unit and at 1- and 6-month follow-up. Clin Psychol.

[79] Symon B, Bammann M, Crichton G, Lowings C, Tucsok J. Reducing postnatal depression, anxiety and stress using an infant sleep intervention. BMJ Open. 2012;2(5):e001662. 10.1136/bmjopen-2012-001662 PMC3467591 22983788

[80] Butler AC, Chapman JE, Forman EM, Beck AT (2006). The empirical status of cognitive-behavioral therapy: a review of meta-analyses. Clin Psychol Rev.

[81] Troy NW, Dalgas-Pelish P (2003). The effectiveness of a self-care intervention for the management of postpartum fatigue. Appl Nurs Res.

[82] Badr H, Zauszniewski J (2017). Meta-analysis of the predictive factors of postpartum fatigue. Appl Nurs Res.

[83] Coo Calcagni S, Bei B, Milgrom J, Trinder J (2012). The relationship between sleep and mood in first-time and experienced mothers. Behav Sleep Med.

[84] Kline P. Handbook of psychological testing. London: Routledge; 2013.

[85] Parkitny L, McAuley JH, Walton D, Pena Costa LO, Refshauge KM, Wand BM, Di Pietro F, Moseley GL (2012). Rasch analysis supports the use of the depression, anxiety, and stress scales to measure mood in groups but not in individuals with chronic low back pain. J Clin Epidemiol.

[86] Shea TL, Tennant A, Pallant JF (2009). Rasch model analysis of the depression, anxiety and stress scales (DASS). BMC Psychiatry.

[87] Åkerstedt T, Axelsson J, Lekander M, Orsini N, Kecklund G (2013). The daily variation in sleepiness and its relation to the preceding sleep episode--a prospective study across 42 days of normal living. J Sleep Res.

[88] Highet NJ, Gemmill AW, Milgrom J (2011). Depression in the perinatal period: awareness, attitudes and knowledge in the Australian population. Aust N Z J Psychiatry.

[89] Fallon V, Halford JCG, Bennett KM, Harrold JA (2016). The postpartum specific anxiety scale: development and preliminary validation. Arch Womens Ment Health.

[90] Giallo R, Wade C, Kienhuis M (2014). Fatigue in mothers of infants and young children: factor structure of the fatigue assessment scale. Fatigue Biomed Health Behav.

[91] Somerville S, Dedman K, Hagan R, Oxnam E, Wettinger M, Byrne S, Coo S, Doherty D, Page AC (2014). The perinatal anxiety screening scale: development and preliminary validation. Arch Womens Ment Health.

[92] Wilson N, Wynter K, Fisher J, Bei B. Postpartum fatigue: assessing and improving the psychometric properties of the fatigue severity scale. Archives of Women's Mental Health. 2018;21(4):471-74. 10.1007/s00737-018-0818-1 29455254

[93] Ballesio A, Aquino MRJV, Feige B, Johann AF, Kyle SD, Spiegelhalder K, Lombardo C, Rücker G, Riemann D, Baglioni C. The effectiveness of behavioural and cognitive behavioural therapies for insomnia on depressive and fatigue symptoms: a systematic review and network meta-analysis. Sleep Med Rev. 2018;37:114-29. 10.1016/j.smrv.2017.01.006 28619248

[94] Woolhouse H, Mercuri K, Judd F, Brown SJ (2014). Antenatal mindfulness intervention to reduce depression, anxiety and stress: a pilot randomised controlled trial of the MindBabyBody program in an Australian tertiary maternity hospital. BMC Pregnancy Childbirth.

[95] Wynter K, Wilson N, Thean P, Bei B, Fisher J. Psychological distress, alcohol use, fatigue, sleepiness, and sleep quality: an exploratory study among men whose partners are admitted to a residential early parenting service. Aust Psychol. 2018.

