# Supplementary material for: More than depression: a multi-dimensional assessment of postpartum distress symptoms before and after a residential early parenting program
Source: BMC Psychiatry. 2019 Jan 29;19:48. doi: 10.1186/s12888-019-2024-8 (PMC6352433; doi:10.1186/s12888-019-2024-8)
Supplement: Supplementary file 1 — Table S1. Pearson Correlations for Self-Report Variables at T1 and T2 (N = 78). (DOC 47 kb) [file 12888_2019_2024_MOESM1_ESM.doc]

**Supplementary Table**

*Pearson Correlations for Self-Report Variables at T1 and T2 (N = 78*)

|  | 1 | 2 | 3 | 4 | 5 | 6 | 7 | 8 | 9 | 10 | 11 | 12 | 13 | 14 |
| --- | --- | --- | --- | --- | --- | --- | --- | --- | --- | --- | --- | --- | --- | --- |
| 1. Maternal age | -- |  |  |  |  |  |  |  |  |  |  |  |  |  |
| 2. Infant age | .28 | -- |  |  |  |  |  |  |  |  |  |  |  |  |
| 3. Infant birth weight | -.08 | -.12 | -- |  |  |  |  |  |  |  |  |  |  |  |
| 4. T1 DASS-D | -.57* | -.20 | -.36 | -- |  |  |  |  |  |  |  |  |  |  |
| 5. T1 DASS-A | -.63* | -.53* | -.27 | .72** | -- |  |  |  |  |  |  |  |  |  |
| 6. T1 DASS-S | -.41 | -.44 | -.51 | .72** | .70** | -- |  |  |  |  |  |  |  |  |
| 7. T1 IDA-I | -.42 | -.13 | -.57* | .80** | .60* | .83** | -- |  |  |  |  |  |  |  |
| 8. T1 FSS | -.17 | -.25 | -.31 | .41 | .24 | .67** | .63* | -- |  |  |  |  |  |  |
| 9. T1 ISI | -.44 | -.35 | -.56* | .41 | .45 | .72** | .53* | .50 | -- |  |  |  |  |  |
| 10. T2 DASS-D | -.40 | -.14 | -.28 | .64* | .44 | .20 | .38 | -.10 | .00 | -- |  |  |  |  |
| 11. T2 DASS-A | -.20 | -.42 | -.10 | -.06 | .40 | .02 | -.08 | -.35 | .00 | .39 | -- |  |  |  |
| 12. T2 DASS-S | -.26 | -.20 | -.05 | .23 | .25 | -.03 | .03 | -.35 | -.06 | .77** | .59* | -- |  |  |
| 13. T2 IDA-I | -.34 | -.12 | -.20 | .42 | .27 | -.00 | .23 | -.27 | -.07 | .90** | .38 | .77** | -- |  |
| 14. T2 FSS | .09 | -.03 | -.10 | -.04 | -.25 | -.12 | .04 | .11 | -.18 | .26 | -.03 | .19 | .37 | -- |
| 15. T2 ISI | -.24 | -.30 | -.41 | .18 | .20 | .09 | .14 | -.16 | .34 | .60* | .51 | .68** | .68** | .38 |

*Note.* * *p* < .05, ** *p* < .01. DASS-D, DASS-A, and DASS-S are Depression Anxiety Stress Scale-21 Depression, Anxiety and Stress subscales respectively; FSS = Fatigue Severity Scale; IDA-I = Irritability Depression Anxiety Scale – Irritability Subscale; ISI = Insomnia Severity Index.
